# Tirzepatide as Adjunct to Insulin in Adults With Type 1 Diabetes and Overweight or Obesity: A Systematic Review of Randomized and Real‐World Evidence

**DOI:** 10.1002/edm2.70225

**Published:** 2026-04-20

**Authors:** Giuseppina Alessia Acucella, Danilo Caponio

**Affiliations:** ^1^ Dipartimento di Medicina di Precisione e Rigenerativa e Area Jonica Universita Degli Studi di Bari Aldo Moro Bari Italy

## Abstract

**Background:**

Overweight and obesity are increasingly common in adults with type 1 diabetes (T1D), contributing to insulin resistance, higher insulin requirements, and greater cardiometabolic burden. Tirzepatide, a dual glucose‐dependent insulinotropic polypeptide and glucagon‐like peptide‐1 receptor agonist, has shown major metabolic benefits in type 2 diabetes and obesity, but its role in T1D remains unclear. This systematic review evaluated tirzepatide as adjunctive therapy to insulin in adults with T1D and overweight or obesity.

**Methods:**

This review followed PRISMA 2020 and was registered in PROSPERO (CRD420261335230). PubMed/MEDLINE, Embase, Scopus, Web of Science Core Collection, and ClinicalTrials.gov were searched from inception to March 1, 2026. Eligible studies included randomized and observational studies reporting efficacy or safety outcomes of tirzepatide added to insulin in adults with T1D. Because of marked clinical and methodological heterogeneity, findings were synthesized qualitatively without meta‐analysis, and certainty of evidence was assessed using a GRADE‐based framework.

**Results:**

Eight studies were included: one small 12‐week phase 2 randomized placebo‐controlled trial and seven observational studies, most at serious risk of bias. The most consistent finding was body weight reduction. In the randomized trial, tirzepatide reduced mean body weight by 10.3 kg, with an estimated treatment difference of 8.7 kg versus placebo, corresponding to an 8.8% reduction from baseline. A placebo‐adjusted 35.1% reduction in total daily insulin dose and a between‐group HbA1c difference of −0.4 percentage points were also reported, although glycaemic findings were short‐term and imprecise. Gastrointestinal adverse events were the most frequent safety findings. Evidence certainty was low or very low.

**Conclusions:**

Tirzepatide may be a promising investigational adjunct in selected adults with T1D and overweight or obesity, particularly for weight reduction. However, current evidence remains insufficient to establish durable glycaemic benefit or long‐term safety. Larger randomized trials are needed.

## Graphical abstract

Tirzepatide as adjunct to insulin in adults with type 1 diabetes and overweight or obesity shows the most consistent signal for body weight reduction, with possible improvements in HbA1c and insulin requirements. However, current evidence remains early, low‐certainty, and insufficient to establish long‐term efficacy or safety.

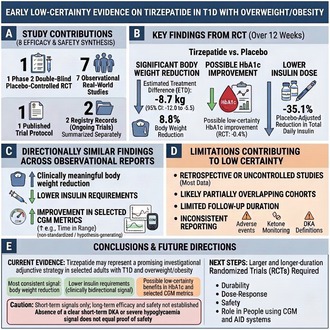

## 1 Introduction

The clinical phenotype of type 1 diabetes (T1D) has evolved substantially over recent decades. Once traditionally associated with a lean phenotype, T1D is now increasingly accompanied by overweight or obesity. This shift reflects broader population‐level trends as well as diabetes‐specific contributors, including intensive insulin therapy, defensive eating related to hypoglycaemia prevention, reduced physical activity, and the cumulative metabolic burden of chronic disease management [1, 2]. Excess adiposity in T1D is clinically relevant because it is associated with insulin resistance, higher insulin requirements, greater glycaemic variability, and a less favourable cardiometabolic risk profile [2].

Despite major advances in insulin therapy, continuous glucose monitoring (CGM), and automated insulin delivery (AID) systems, a substantial proportion of adults with T1D still do not achieve recommended glycaemic targets [3]. Intensification of insulin therapy alone may improve glucose levels but often at the cost of additional weight gain, greater treatment complexity, and increased risk of hypoglycaemia. This creates a self‐reinforcing cycle in which escalating insulin doses may worsen insulin resistance and weight‐related metabolic burden. In this context, adjunctive therapies capable of reducing insulin requirements while improving metabolic efficiency are of considerable clinical interest.

Non‐insulin adjunctive strategies in T1D have yielded mixed results. Sodium‐glucose cotransporter‐2 inhibitors demonstrated metabolic benefits in selected settings but raised substantial concerns regarding diabetic ketoacidosis (DKA), limiting widespread clinical adoption [4]. GLP‐1 receptor agonists have also been evaluated in T1D and appear to produce clinically relevant reductions in body weight and insulin dose, whereas glycaemic benefits have varied across studies and may depend on baseline adiposity, residual endogenous insulin secretion, and use of diabetes technologies [5]. More recent semaglutide studies in adults with T1D and obesity suggest that benefit may extend beyond weight reduction and include improvement in CGM‐based glycaemic outcomes, particularly in technology‐supported populations [6, 7].

Tirzepatide is a dual glucose‐dependent insulinotropic polypeptide (GIP) and glucagon‐like peptide‐1 (GLP‐1) receptor agonist that has shown greater weight loss and broad metabolic effects in type 2 diabetes and obesity than earlier incretin‐based therapies [8]. Because individuals with T1D were excluded from pivotal tirzepatide trials, evidence in T1D has only recently begun to emerge. The currently available randomized evidence consists of a single 12‐week phase 2 placebo‐controlled trial in adults with T1D and obesity using low‐dose tirzepatide, 2.5 mg weekly for 4 weeks followed by 5 mg weekly for 8 weeks [9]. Real‐world evidence is based largely on retrospective cohorts and comparative observational analyses [10, 11, 12, 13, 14, 15, 16]. Larger dedicated studies are underway, including the published TZP‐T1D protocol and sponsor‐led trials registered as NCT06914895 and NCT06962280 [17, 18, 19].

A brief mechanistic context is important. In established T1D, any benefit of tirzepatide is unlikely to depend primarily on enhancement of endogenous insulin secretion. More plausible pathways include appetite suppression, reduced caloric intake, delayed gastric emptying, reduction in adiposity, and improvement in insulin sensitivity. GIP‐related effects on glucagon regulation, adipose tissue metabolism, or nutrient handling may also be relevant, but their specific contribution in T1D, particularly in individuals with minimal or absent endogenous insulin secretion, remains uncertain. Thus, mechanistic plausibility exists but should not be overstated in the absence of definitive clinical evidence.

The aim of the present systematic review was not to provide therapeutic recommendations, but to critically summarize the currently available evidence, distinguish what can reasonably be inferred from what remains uncertain, formally appraise the certainty of evidence, and identify key research priorities regarding tirzepatide use in T1D.

## 2 Methods

### 2.1 Study Design and Reporting Framework

This systematic review was conducted and reported in accordance with the Preferred Reporting Items for Systematic Reviews and Meta‐Analyses (PRISMA) 2020 statement [20]. A protocol for this review was registered in PROSPERO (CRD420261335230). Given the early stage of the evidence base, with only one randomized controlled trial and several heterogeneous non‐randomized studies, the review was designed a priori as a qualitative synthesis rather than a quantitative meta‐analysis.

The review question, eligibility criteria, prespecified outcomes, risk‐of‐bias framework, certainty‐of‐evidence approach, and decision not to perform quantitative pooling in the presence of marked clinical and methodological heterogeneity were defined prospectively and documented in the review protocol.

### 2.2 Search Strategy

A comprehensive literature search was performed in PubMed/MEDLINE, Embase (Ovid), Scopus, Web of Science Core Collection, and ClinicalTrials.gov from database inception to March 1, 2026. The search strategy combined controlled vocabulary and free‐text terms for type 1 diabetes and tirzepatide, including alternate developmental and product naming conventions such as LY3298176, Mounjaro, and Zepbound where applicable. To maximize sensitivity, the core search structure was based on the intersection of the population concept (type 1 diabetes) and the intervention concept (tirzepatide), without requiring additional outcome‐ or phenotype‐related terms such as obesity, insulin dose, or adjunctive therapy. Database‐specific syntax was adapted for each source, and the full search strategies are reported in Appendix S1.

Searches were supplemented by manual screening of reference lists from eligible studies, relevant reviews, and trial protocols. Only English‐language full‐text articles were included in the final synthesis. This restriction was adopted for feasibility; however, language bias cannot be excluded.

### 2.3 Eligibility Criteria

Eligible studies met all of the following criteria:

1. Adults with T1D treated with tirzepatide in addition to insulin;
2. Randomized controlled trials, prospective observational studies, retrospective cohort studies, or comparative real‐world studies;
3. Reporting at least one prespecified efficacy or safety outcome

The following were excluded from the efficacy synthesis: single‐patient case reports, case‐based publications without extractable cohort data, conference abstracts without sufficient methodological detail, review articles, editorials, comments, and letters without original outcome data. Published trial protocols and ClinicalTrials.gov records were retained only to describe the evolving evidence base and were not considered outcome‐bearing studies.

### 2.4 Outcomes

The prespecified primary outcomes were change in body weight and change in HbA1c. Secondary outcomes included change in total daily insulin dose, changes in CGM‐derived metrics including time in range (TIR), time above range (TAR), time below range (TBR), and mean sensor glucose, as well as safety outcomes including DKA, ketosis, severe hypoglycaemia, gastrointestinal adverse events, treatment discontinuation, and serious adverse events.

Because CGM metrics were non‐uniformly defined and reported across studies, these outcomes were prespecified as exploratory and interpreted as non‐standardized, hypothesis‐generating measures rather than as directly comparable endpoints.

### 2.5 Study Selection and Management of Potential Overlap

Two reviewers independently screened titles and abstracts, followed by full‐text review of potentially eligible reports. Disagreements were resolved by discussion and consensus. When multiple studies appeared to arise from the same center, investigative network, or partially overlapping recruitment periods, reports were examined specifically for possible overlap in patient populations. In such cases, the studies were interpreted as complementary analyses rather than fully independent confirmatory datasets unless cohort independence was clearly demonstrated. A structured assessment of possible non‐independence across observational reports is provided in Appendix S2.

### 2.6 Data Extraction

Data were extracted using a standardized form capturing study design and setting, sample size, participant characteristics, baseline body weight, baseline body mass index (BMI), baseline HbA1c, insulin modality, technology use, tirzepatide dosing and escalation, duration of follow‐up, efficacy outcomes, safety outcomes, and methodological limitations.

To facilitate clinical interpretation, body weight changes were preferentially reported in both absolute and relative terms when available. HbA1c values were reported in percentage units, with any standardized conversions to be provided in tabular form where required by journal style.

For safety, particular attention was paid to whether adverse events were actively monitored or passively reported, whether ketone testing was protocolized or discretionary, whether DKA definitions were explicitly stated, and whether severe hypoglycaemia and discontinuation were systematically captured.

### 2.7 Risk of Bias Assessment

Risk of bias was assessed using the Cochrane Risk of Bias 2 (RoB 2) tool for randomized trials and ROBINS‐I for non‐randomized studies [21, 22]. Particular attention was paid to confounding by indication, participant selection, missing outcome data, inconsistent adverse‐event ascertainment, possible overlap across institutional cohorts, and bias arising from non‐standardized insulin titration.

For observational studies, the ROBINS‐I domains most commonly driving a serious overall judgement were confounding, selection of participants into the study, missing data, and measurement of outcomes, particularly for safety‐related endpoints. A structured summary is presented in Table 4.

### 2.8 Certainty of Evidence

Certainty of evidence for key outcomes was assessed using a GRADE‐based framework. A Summary of Findings is provided in (Appendix S4). The main outcomes evaluated for certainty were body weight reduction, HbA1c, total daily insulin dose, CGM‐derived metrics, severe hypoglycaemia, DKA/ketosis, gastrointestinal adverse events, and treatment discontinuation.

Given the evidence base, consisting of one small short‐duration randomized trial with some concerns and multiple observational studies at serious risk of bias, certainty was anticipated to be low or very low across most outcomes.

### 2.9 Statistical Methods

No quantitative meta‐analysis was performed. Heterogeneity was considered too substantial to support meaningful pooled effect estimation because studies differed in design, inclusion criteria, baseline adiposity, insulin delivery modality, tirzepatide exposure and dose escalation, comparator choice, duration of follow‐up, definitions and completeness of safety outcomes, and reporting of CGM metrics and insulin dose changes. In addition, possible partial overlap among some observational cohorts reduced confidence in the independence assumptions required for pooling.

Pooling was also considered potentially misleading because large effect estimates from selected real‐world cohorts may have been amplified by selection and attrition, and because effect estimates for glycaemic outcomes and insulin reduction may reflect a mixture of direct drug effect, mediated weight‐loss effects, technology use, and clinician‐driven insulin retitration.

Accordingly, randomized evidence was summarized descriptively and observational evidence was synthesized narratively.

## 3 Results

### 3.1 Study Selection

**FIGURE 1:**
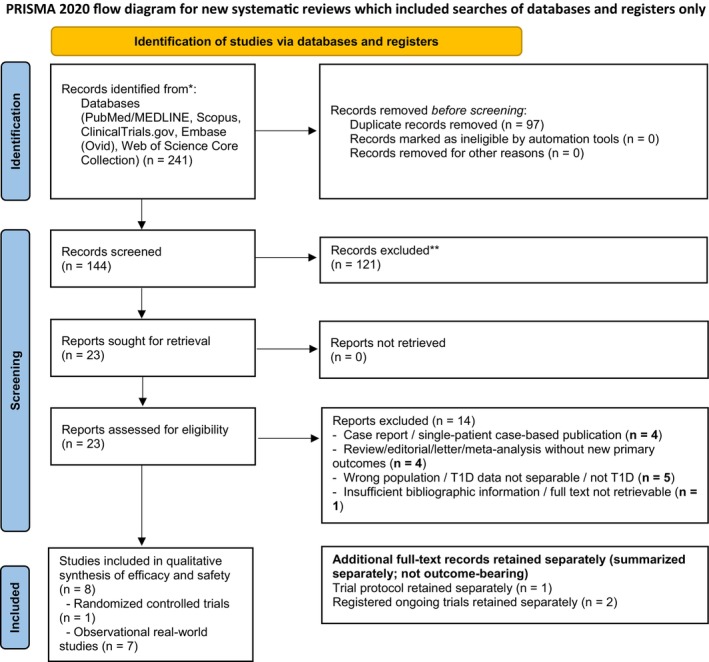
PRISMA 2020 flow diagram for new systematic reviews which included searches of databases and registers only.

The database and registry search identified 241 records. After duplicate removal, 144 records underwent title and abstract screening. Twenty‐three full‐text reports were assessed for eligibility. Eight studies were included in the efficacy and safety synthesis. In addition, one published trial protocol and two ClinicalTrials.gov registry records were retained separately to describe ongoing evidence generation and were not considered outcome‐bearing studies. Fourteen full‐text reports were excluded with reasons, which are detailed in the PRISMA flow diagram: Figure 1, Table 1, and Appendix S3.

**TABLE 1:** PRISMA study selection summary.

PRISMA stage	Number
Records identified through database and registry searching	241
Duplicate records removed	97
Records screened by title and abstract	144
Records excluded (title/abstract)	121
Full‐text reports assessed for eligibility	23
Full‐text reports excluded (with reasons)	14
Studies included in efficacy and safety synthesis	8
Trial protocol retained separately (no outcomes)	1
Registered ongoing trials summarized separately	2

*Note:* Summary of the study identification and selection process for this systematic review. The database and registry search identified 241 records. After duplicate removal, 144 records underwent title and abstract screening. Twenty‐three full‐text reports were assessed for eligibility. Eight studies met criteria for inclusion in the qualitative synthesis of efficacy and safety, comprising one randomized controlled trial and seven observational real‐world studies. Fourteen full‐text reports were excluded with protocol‐defined reasons. One published trial protocol and two registered ongoing trials were retained separately to describe the evolving evidence base and were not considered outcome‐bearing studies.

### 3.2 Characteristics of Included Studies

The included efficacy studies comprised one phase 2 randomized placebo‐controlled trial and seven observational studies, most of which were retrospective and conducted in specialty diabetes centers. Study populations were enriched for overweight or obesity, with baseline BMI commonly in the overweight‐to‐obese range and, in several cohorts, severe obesity. Baseline HbA1c generally ranged from approximately 7.0%–8.5%, and follow‐up ranged from 12 weeks to 21 months. Tirzepatide exposure varied from low‐dose initiation in the randomized study to real‐world dose escalation according to tolerability and clinician preference in observational cohorts. Several studies originated from the same or related investigative networks and may have included partially overlapping patient populations.

The current evidence base is therefore best understood as one small randomized proof‐of‐concept trial plus a series of observational reports that are informative but methodologically fragile and not always fully independent. Key study characteristics are summarized in Table 2.

**TABLE 2:** Characteristics of included studies evaluating tirzepatide in adults with type 1 diabetes.

Study	Design	Population/sample	Baseline weight/BMI/HbA1c	Follow‐up	Tirzepatide exposure	Main efficacy findings	Main limitations
Snaith et al. [9]	Phase 2 randomized double blind placebo controlled trial	Adults with T1D and obesity; 24 randomized, 22 completed	Mean weight 101 ± 14 kg, mean BMI 33.7 ± 3 kg/m^2^, mean HbA1c 7.3% ± 1.2% (tirzepatide vs. placebo: 104.8 ± 14.6 kg vs. 97.7 ± 12.9 kg; BMI 34.2 ± 3.4 vs. 33.2 ± 3.0; HbA1c 7.3% ± 1.3% vs. 7.2% ± 1.2%)	12 weeks	2.5 mg weekly for 4 weeks, then 5 mg weekly for 8 weeks	−10.3 kg vs. −0.7 kg; ≈8.8% weight reduction; HbA1c difference −0.4 percentage points; insulin dose reduction −35%	Small sample; short duration; low dose; underpowered for rare events
Akturk et al. [10]	Proof of concept observational cohort	Adults with T1D; *n* = 26	Mean weight 108.1 ± 21.2 kg, mean BMI 36.7 ± 5.3 kg/m^2^, mean HbA1c 7.3% ± 0.7%	Up to 8 months	Real‐world use	HbA1c reduction of −0.45% at 3 months and −0.59% at 8 months; weight −3.4%, −10.5%, −10.1% at 3, 6, 8 months; TIR +12.6% at 3 months	Uncontrolled; small sample; selection bias; limited safety data
Garg et al. [11]	Matched comparative observational study	Adults with T1D and overweight/obesity; 62 treated vs. 37 controls	Not reported	12 months	Real‐world use; dose escalation per care pattern	Mean weight loss 18.5% at 1 year; improvements in HbA1c, insulin dose, mean glucose, TIR and TAR	Not randomized; residual confounding; treated subset selected from wider cohort; potential survivor bias
Karakus et al. [12]	Observational cohort of AID users	Adults with T1D using Tandem Control IQ; *n* = 11	Median weight 114.3 kg (IQR 94.8–129.3), median BMI 39.6 kg/m^2^ (35.6–40.7), median HbA1c 7.0% (6.7–7.4)	8 months	Real‐world use	Total insulin dose −30% within 2 months (basal −31%, bolus −43%), HbA1c −0.5%, TIR +7%, weight −9%	Very small sample; device specific cohort; nonrandomized; possible overlap with other Colorado cohorts
Rivera Gutierrez et al. [13]	Retrospective observational cohort	Adults with T1D and overweight/obesity; *n* = 51	Median weight 96.9 kg; median BMI 34.9 kg/m^2^; median HbA1c 8.0%	Median 8 months (subset to 12 months)	Real‐world use	Median weight reduction 8.5% at ~8 months and 12.2% at 12 months; HbA1c −0.9%; insulin requirement −31.6%	Retrospective design; incomplete long term follow‐up; single center cohort
SnellBergeon et al. [14]	Real‐world comparative study	Adults with T1D and overweight/obesity treated with semaglutide or tirzepatide	Not reported	12 months	Real‐world use	Tirzepatide: −49.4 ± 3.0 lb. (≈−22.4 kg; −21.4%); HbA1c −0.68% ± 0.16%; controls showed no meaningful change	Observational design; treatment selection bias; possible cohort nonindependence
Garg et al. [15]	Longterm matched observational analysis	Adults with T1D and overweight/obesity; *n* = 84 treated	Not reported	21 months	Real‐world use	Weight −59 ± 4.6 lb. (≈−26.8 kg; −23.4%) vs. weight gain in controls; HbA1c reduction; favorable cardiometabolic biomarkers	Observational design; survivor bias; possible overlap with related Colorado cohorts; biomarker focused
Al Ozairi et al. [16]	Real‐world comparative study	Adults with T1D and BMI ≥ 27 kg/m^2^; about 250 participants	BMI ≥ 27 kg/m^2^ (weight and HbA1c not reported)	12 months	Tirzepatide vs. semaglutide vs. liraglutide	Weight change: −10.9% vs. −9.9% vs. −7.1%; HbA1c change: −0.65% vs. −0.33% vs. −0.23%	Nonrandomized allocation; residual confounding; safety not adjudicated

*Note:* Overview of study design, population, baseline characteristics, follow‐up duration, tirzepatide exposure, main efficacy findings, and major limitations of the included randomized and observational studies.

Abbreviations: “Not reported”, not provided in accessible sources; TAR, time above range; TIR, time in range.

Where explicitly reported, baseline body weight was generally approximately 100–114 kg, baseline BMI ranged from approximately 33.7–39.6 kg/m^2^, and baseline HbA1c from approximately 7.0%–8.0%, consistent with overweight/obesity‐enriched populations.

### 3.3 Randomized Evidence

The only randomized study identified was a phase 2, double‐blind, placebo‐controlled trial conducted over 12 weeks in adults with T1D and obesity [9]. Twenty‐two of 24 randomized participants completed the study. Tirzepatide produced a significantly greater reduction in body weight than placebo, with a mean change of −10.3 kg (95% confidence interval [CI], −12.8 to −7.7) in the tirzepatide group versus −0.7 kg in the placebo group, yielding an estimated treatment difference of −8.7 kg (95% CI, −12.0 to −5.5; *p* < 0.0001), corresponding to an 8.8% body weight reduction [9]. All tirzepatide‐treated participants achieved at least 5% weight loss and 45% achieved at least 10% weight loss, compared with 9% and 0%, respectively, in the placebo group [9].

Tirzepatide was also associated with a between‐group difference in HbA1c of −0.4 percentage points (95% CI, −0.7 to 0.0; *p* = 0.05) and a placebo‐adjusted reduction in total daily insulin dose of 35.1% (95% CI, −46.5 to −21.3; *p* = 0.0002) [9]. In adults with T1D, an HbA1c reduction of this magnitude may be clinically meaningful; however, the result remains short‐term and imprecise and should not be interpreted as establishing a durable or independent glycaemic effect.

Safety findings from the randomized trial require careful interpretation. No DKA, severe hypoglycaemia, or serious adverse events were reported during the 12‐week study period; gastrointestinal adverse events consistent with the incretin class profile were reported [9]. However, the trial was small, brief, and limited to low‐dose tirzepatide. It was not structurally capable of informing uncommon adverse events or long‐term safety.

### 3.4 Observational Real‐World Evidence

Observational studies generally suggested that tirzepatide may produce clinically meaningful body weight reduction in adults with T1D, especially in populations enriched for overweight or obesity [10, 11, 12, 13, 14, 15, 16]. The body‐weight signal was the most consistent finding across studies, whereas glycaemic and CGM outcomes were more variable and more vulnerable to confounding.

In the 26‐patient proof‐of‐concept cohort reported by Akturk et al., HbA1c fell by 0.45% at 3 months and 0.59% at 8 months, while body weight declined by 3.4%, 10.5%, and 10.1% at 3, 6, and 8 months, respectively [10]. TIR increased by 12.6 percentage points and TAR decreased by 12.6 percentage points at 3 months, with improvements reportedly maintained through 8 months [10]. One severe hypoglycaemia event occurred during follow‐up, and two patients discontinued therapy [10].

In the matched comparative study by Garg et al., tirzepatide was associated with marked weight reduction over 1 year together with improvements in HbA1c, insulin dose, and selected CGM metrics [11]. However, the treated cohort represented a selected subset of patients prescribed tirzepatide, already indicating potential selection and survivor bias. Thus, while the findings support a strong apparent body‐weight signal, the possibility of effect overestimation due to patient selection, attrition, and non‐randomized care patterns must be acknowledged.

In adults using AID, Karakus et al. reported an approximately 30% reduction in total daily insulin dose within 2 months of tirzepatide initiation, sustained over 8 months [12]. TIR improved, HbA1c declined, and body weight decreased. However, this was a very small, device‐specific, non‐randomized cohort, and the authors noted that the analysis used data derived from a previously published tirzepatide study, reinforcing concerns about non‐independence across reports.

Additional real‐world data from Mayo Clinic supported these observations. In a retrospective study of adults with T1D and overweight or obesity, total body‐weight loss increased over follow‐up, with parallel reductions in HbA1c and insulin requirements [13]. Comparative real‐world studies likewise suggested substantial body‐weight reduction with tirzepatide relative to other incretin‐based therapies or matched controls [14, 15, 16]. Some of the largest reported reductions exceeded 20% over 12–21 months [14, 15]. Although clinically striking, such estimates should be interpreted cautiously because observational selection, attrition, survivor bias, and preferential retention of adherent or treatment‐responsive individuals may inflate apparent benefit.

Overall, the observational literature shows directionally similar findings in body weight, insulin requirements, and selected CGM metrics. However, this apparent consistency should not be overinterpreted as independent replication. Most real‐world studies were retrospective or uncontrolled; all non‐randomized studies were at serious risk of bias, insulin adjustment was not standardized, follow‐up intensity varied, and several reports appear to derive from the same or related clinical and investigative networks and may include partially overlapping patient populations. Once non‐independence is acknowledged, the perceived robustness of the real‐world signal is substantially attenuated.

### 3.5 Interpretation of Insulin Dose Reduction

Across studies, reductions in insulin dose were common. This finding is clinically important, but it is not a unidirectionally beneficial endpoint in T1D. Lower insulin dose may reflect improved metabolic efficiency, reduced adiposity, or reduced insulin resistance. At the same time, insulin dose is also a direct mediator of glycaemic control and a critical safeguard against ketosis. Excessive reduction, particularly in the setting of reduced carbohydrate intake, nausea, vomiting, dehydration, intercurrent illness, or overaggressive dose adjustment, may contribute to harm.

Insulin dose reduction should therefore be interpreted as a bidirectional clinical signal rather than a purely favorable outcome. Its interpretation must be linked to the absence of standardized insulin titration protocols in the available literature, the possibility of under insulinization, and the biological plausibility of increased ketogenesis risk.

The current data also do not establish whether tirzepatide exerts a truly insulin‐sparing effect independent of weight loss. In many reports, declining insulin dose appears to track with declining body weight, suggesting mediation by reduced adiposity and improved insulin sensitivity. In other instances, insulin reduction appears early and may also reflect reduced caloric intake, increased clinical attention, or proactive clinician‐driven titration. With the existing evidence, these pathways cannot be disentangled.

### 3.6 Glycaemic Outcomes

The available literature suggests a glycaemic benefit signal, but not yet a robust one. In the randomized trial, the between‐group HbA1c difference was −0.4 percentage points over 12 weeks [9]. This magnitude may be clinically meaningful in T1D, but it remains a short‐term finding from a very small sample with borderline statistical precision. In observational studies, HbA1c improvements generally ranged from approximately 0.4%–0.9% [10, 11, 12, 13, 14, 15, 16], and several reports described improvements in CGM‐derived metrics.

These findings are plausible and should not be dismissed. However, they should also not be interpreted as evidence of a stable, independent glycaemic effect of tirzepatide. In observational studies, glycaemic improvements may reflect body‐weight reduction, changes in insulin titration, increased clinical attention, use of CGM/AID systems, and selection or adherence bias. CGM outcomes, in particular, were non‐standardized across studies and should therefore be regarded as hypothesis‐generating rather than definitive.

### 3.7 Safety and Tolerability

Across included studies, gastrointestinal intolerance was the most commonly reported adverse‐event domain, consistent with the established incretin class profile [9, 10, 11, 12, 13, 14, 15, 16]. Short‐term severe hypoglycaemia and DKA were not reported as clearly increased in the currently available literature. However, this should not be interpreted as demonstration of safety. A structured synthesis of safety findings is presented in Table 3.

**TABLE 3:** Structured safety synthesis across included studies.

Study	Gastrointestinal adverse events	Severe hypoglycaemia	DKA/ketosis	DKA definition prespecified?	Ketone monitoring protocolized?	Discontinuation reporting	Safety interpretation
Snaith et al. [9]	Reported; consistent with incretin class profile	No events reported	No events reported	Not clearly specified in available summary	Not clearly described in available summary	Limited by short follow‐up	Randomized evidence is informative for short‐term tolerability, but the trial was underpowered for uncommon harms such as DKA or severe hypoglycaemia
Akturk et al. [10]	Reported	1 event reported	No events reported	Not specified	Not standardized	Reported, but incompletely detailed	Safety ascertainment was observational and non‐standardized; absence of reported DKA does not exclude clinically relevant risk
Garg et al. [11]	Reported	No hospitalization for severe hypoglycaemia reported	No hospitalization for DKA reported	Not specified	Not standardized	Incompletely detailed	Hospitalization‐level reporting is not equivalent to systematic adverse‐event ascertainment and may underestimate event burden
Karakus et al. [12]	Not a major focus of reporting	Not specifically reported	No events reported	Not specified	Not protocolized	Not systematically detailed	Very small AID‐specific cohort with limited ability to detect uncommon adverse events; no validated safety inference can be made
Rivera Gutierrez et al. [13]	Reported, including nausea	No worsening in hypoglycaemia reported	No events reported	Not specified	Not standardized	Limited retrospective reporting	Retrospective chart review limits completeness of adverse‐event capture, severity grading, and event adjudication
Snell‐Bergeon et al. [14]	Reported	Not adjudicated uniformly	No events reported	Not specified	Not protocolized	Limited detail	Comparative observational design is informative descriptively, but under‐ascertainment of DKA and severe hypoglycaemia remains possible
Garg et al. [15]	Variably reported	Not adjudicated prospectively	No events reported	Not specified	Not clearly described	Not uniformly reported	Longer follow‐up is informative, but safety reporting remained observational, non‐standardized, and potentially incomplete
Al Ozairi et al. [16]	Reported; class‐consistent gastrointestinal profile	No events reported	No events reported	Not specified	Not standardized	Real‐world reporting only	Comparative real‐world data are useful descriptively, but do not provide adjudicated reassurance regarding uncommon harms

*Note:* Summary of reported gastrointestinal adverse events, severe hypoglycaemia, DKA/ketosis, DKA definition, ketone monitoring, discontinuation reporting, and overall safety interpretation across included studies. Overall, gastrointestinal adverse events were the most commonly reported safety findings across included studies. However, safety ascertainment was inconsistent, ketone monitoring was generally not protocolized, DKA definitions were rarely specified, and discontinuation reporting was incomplete in several reports. Accordingly, the absence of a clear short‐term signal for DKA or severe hypoglycaemia should be interpreted as absence of evidence rather than evidence of absence. Contextual case reports of severe euglycaemic DKA, particularly in the setting of concomitant SGLT2 inhibitor use, further reinforce the need for cautious interpretation and standardized surveillance in future trials.

Abbreviations: AID, automated insulin delivery; DKA, diabetic ketoacidosis.

The randomized trial enrolled only 24 participants and followed them for 12 weeks [9]. Observational studies generally lacked standardized definitions of DKA, systematic ketone monitoring, active adverse‐event adjudication, and consistent reporting of discontinuation and exposure duration [10, 11, 12, 13, 14, 15, 16]. Thus, the evidence base is structurally underpowered and methodologically underdeveloped for reassurance regarding uncommon but clinically consequential harms.

This distinction is particularly important in T1D. A therapy that reduces appetite, caloric intake, body weight, and insulin requirements may create a setting in which under insulinization and ketogenesis become more likely, especially during intercurrent illness, reduced carbohydrate intake, dehydration, or overly aggressive insulin reduction. Therefore, the current literature does not establish a major short‐term safety signal, but neither does it provide sufficiently rigorous evidence to exclude clinically meaningful risk. In this context, the absence of a clear safety signal should be interpreted strictly as absence of evidence, not evidence of absence.

Case reports, while excluded from the efficacy synthesis, remain clinically relevant warning signals. One case report described successful off‐label tirzepatide use in a patient with T1D and obesity, whereas another reported severe euglycaemic DKA in a patient with T1D receiving tirzepatide together with an SGLT2 inhibitor [23, 24]. Additional case literature describes euglycaemic ketoacidosis with tirzepatide plus SGLT2 inhibition in diabetes more broadly, reinforcing biological plausibility even if not all reports are specific to T1D [25].

### 3.8 Risk of Bias

The randomized trial was judged to have some concerns overall under RoB 2. The main limitations relate to imprecision and brief follow‐up rather than to a major internal validity flaw.

All observational studies were judged to have at least serious risk of bias under ROBINS‐I because of confounding, participant selection, incomplete follow‐up, non‐standardized insulin adjustment, inconsistent adverse‐event ascertainment, and possible partial overlap between institutional cohorts. The ROBINS‐I domains most strongly driving serious judgements were confounding, selection of participants, missing data, and measurement of outcomes. These judgements are summarized in Table 4.

**TABLE 4:** Risk‐of‐bias summary.

Study	Tool	Main concerns driving judgement	Overall judgement
Snaith et al. [9]	RoB 2	Small sample and short follow‐up limit precision and generalizability; no major obvious flaw in design, but limited duration constrains inference on durability and safety	Some concerns
Akturk et al. [10]	ROBINS‐I	No comparator; confounding by indication; participant selection; small cohort; incomplete and non‐standardized adverse‐event ascertainment	Serious
Garg et al. [11]	ROBINS‐I	Residual confounding; selection of analysed treated subset from a broader prescribed population; likely survivor bias; non‐standardized insulin adjustment; incomplete safety ascertainment	Serious
Karakus et al. [12]	ROBINS‐I	Very small sample; device‐specific subgroup; no comparator; clinically driven insulin titration; likely overlap with previously published cohort	Serious
Rivera Gutierrez et al. [13]	ROBINS‐I	Retrospective design; incomplete follow‐up; missing long‐term data for some participants; non‐standardized insulin adjustment; outcome measurement limitations for safety endpoints	Serious
Snell‐Bergeon et al. [14]	ROBINS‐I	Treatment‐selection bias; residual confounding; possible cohort non‐independence; observational comparative design without randomized allocation	Serious
Garg et al. [15]	ROBINS‐I	Long‐term observational analysis vulnerable to survivor bias and attrition; biomarker‐focused reporting; possible partial overlap with related treated cohort; non‐standardized adverse‐event reporting	Serious
Al Ozairi et al. [16]	ROBINS‐I	Non‐randomized allocation; residual confounding across treatment groups; real‐world safety outcomes not formally adjudicated	Serious

*Note:* Summary of study‐level risk of bias assessed using RoB 2 for the randomized trial and ROBINS‐I for observational studies. For the observational literature, the ROBINS‐I domains most commonly driving the overall serious judgement were confounding, selection of participants, missing data or incomplete follow‐up, and measurement of outcomes, particularly for safety‐related endpoints. Possible partial overlap among some Colorado‐associated reports further reduced confidence that apparent cross‐study consistency reflected independent confirmation.

### 3.9 Certainty of Evidence

Applying a GRADE‐based framework, certainty of evidence was low or very low across all key outcomes. The body‐weight signal was the most credible, but certainty remained limited by reliance on one small short‐term randomized trial and multiple observational studies at serious risk of bias. Certainty for HbA1c and total daily insulin dose was low to very low because of imprecision, confounding, non‐standardized insulin titration, and indirectness. Certainty for CGM‐derived metrics, severe hypoglycaemia, DKA/ketosis, and several safety outcomes was very low because of sparse randomized evidence, inconsistent reporting, lack of adjudication, and insufficient surveillance. A structured Summary of Findings is provided in Appendix S4.

### 3.10 Ongoing Studies

The pipeline of ongoing evidence is important because the currently published dataset remains small. A published protocol, TZP‐T1D, describes a double‐blind, placebo‐matched randomized controlled trial in adults with T1D and overweight or obesity [17]. In addition, sponsor‐led ClinicalTrials.gov records indicate at least two larger studies evaluating tirzepatide in adults with T1D and obesity or overweight, including NCT06914895 and NCT06962280 [18, 19]. These trials are likely to determine whether the encouraging early signals observed in published studies are durable, generalizable, and safe over longer follow‐up.

## 4 Discussion

This systematic review summarizes the earliest randomized and real‐world evidence regarding tirzepatide as adjunctive therapy to insulin in adults with T1D and overweight or obesity. The overall picture is encouraging but remains methodologically immature. Across study designs, the most consistent signal is clinically meaningful body‐weight reduction [9, 10, 11, 12, 13, 14, 15, 16]. This observation is clinically relevant because many adults with T1D and excess adiposity face a difficult combination of high insulin doses, progressive weight gain, insulin resistance, and persistent metabolic burden despite modern diabetes technologies.

The short‐term randomized trial provides the strongest currently available evidence and supports the conclusion that tirzepatide can reduce body weight over 12 weeks in adults with T1D and obesity [9]. It also suggests that reduced insulin requirements and a potentially clinically meaningful HbA1c improvement may occur in parallel. However, these findings should be interpreted precisely. The available randomized evidence supports a short‐term signal of benefit, not a definitive conclusion regarding long‐term glycaemic efficacy, optimal dose escalation, durability, or uncommon adverse events.

The observational literature broadly aligns with the randomized body‐weight signal, but its interpretation requires restraint [10, 11, 12, 13, 14, 15, 16]. Several studies describe large body‐weight reductions, in some cases exceeding 20% over 12–21 months [14, 15]. Although such findings are clinically striking, they may be vulnerable to overestimation due to selection bias, attrition, survivor bias, and preferential retention of adherent or treatment‐responsive individuals. This caveat is especially important when treated cohorts are drawn from larger populations of patients initially prescribed tirzepatide but only a subset contributes analysable follow‐up.

The apparent coherence of the observational literature is also attenuated by possible partial overlap across cohorts from the same institutional or investigative networks. This point is not merely methodological. If reports are not fully independent, directionally similar findings do not represent true external replication across distinct populations and settings. The observational evidence should therefore be regarded as directionally similar but not fully independent.

From a clinical perspective, the strongest current rationale for tirzepatide in T1D lies in selected adults with overweight or obesity, high insulin requirements, or a more insulin‐resistant phenotype. The available literature does not support extrapolation to lean adults with T1D. This target phenotype remains conceptually attractive but empirically underdefined. No validated patient‐selection criteria currently exist, and it remains uncertain whether obesity alone, high insulin dose alone, residual endogenous insulin secretion, technology use, or other phenotypic features best identify likely responders.

The interpretation of glycaemic benefit requires additional nuance. In this review, the observed HbA1c reduction of approximately 0.4%–0.9% across studies should not be considered trivial in T1D. Such improvements may be clinically meaningful, particularly in patients whose baseline HbA1c is already in the 7.0%–7.5% range. Nonetheless, the evidence remains low‐certainty. The current literature does not demonstrate that tirzepatide exerts a stable glycaemic effect independent of body‐weight reduction, insulin retitration, behavioural changes, or technology‐supported care. A more accurate interpretation is that an early, potentially clinically relevant glycaemic signal exists, but its independence from confounding and mediation remains unproven.

The relationship between insulin reduction and body‐weight reduction is similarly unresolved. Reductions in total daily insulin dose may largely track with declining adiposity and improved insulin sensitivity, but they could also reflect changes in food intake, clinical attention, and proactive dose adjustments. The present evidence base is insufficient to separate these mechanisms, and it does not justify treating insulin reduction as a stand‐alone efficacy marker.

Mechanistic considerations provide plausibility, but not proof. Compared with isolated GLP‐1 receptor agonism, dual GIP/GLP‐1 receptor agonism may offer broader effects on appetite regulation, energy intake, adiposity, insulin sensitivity, and possibly glucagon physiology or adipose tissue biology. However, the metabolic relevance of GIP signalling in established T1D remains uncertain, especially in individuals with minimal endogenous insulin secretion. For this reason, mechanistic discussion should remain supportive rather than determinative.

Dose considerations also deserve emphasis. The only randomized controlled trial used low‐dose tirzepatide, 2.5 mg weekly for 4 weeks followed by 5 mg weekly for 8 weeks [9]. In contrast, real‐world studies frequently included escalation toward 10 mg or higher. It is plausible that higher doses may yield greater metabolic benefits in T1D, as seen in type 2 diabetes and obesity, but this extrapolation is not established. Higher doses may also carry unique tolerability or safety implications in T1D, including greater gastrointestinal intolerance, lower caloric intake, more aggressive insulin down‐titration, and potentially greater vulnerability to ketosis. Current evidence does not define the dose–response relationship or dose‐specific safety profile in T1D.

Safety interpretation deserves particular restraint. The absence of a consistent short‐term signal for severe hypoglycaemia or DKA is reassuring only in a limited descriptive sense. It is not equivalent to proof of safety, especially because ketone surveillance, event adjudication, and adverse‐event capture were not standardized across studies. Any agent that facilitates substantial appetite suppression, body‐weight reduction, and insulin reduction may also increase susceptibility to ketosis if insulin down‐titration is excessive or carbohydrate intake falls during illness or reduced oral intake. Future trials must therefore include protocolized ketone monitoring, prespecified DKA definitions, systematic recording of discontinuation, and transparent reporting of insulin‐adjustment algorithms.

The present review has limitations. Only one randomized trial was available, and it was small and brief. The observational literature was heterogeneous and predominantly retrospective. Several studies may have arisen from overlapping clinical networks, which weakens the inference that consistency across publications reflects independent replication. CGM outcomes and safety outcomes were not reported in a standardized manner, and DKA‐relevant surveillance was usually insufficient. Finally, because of heterogeneity and possible non‐independence across observational cohorts, meta‐analysis would likely have been more misleading than informative.

**FIGURE 2:**
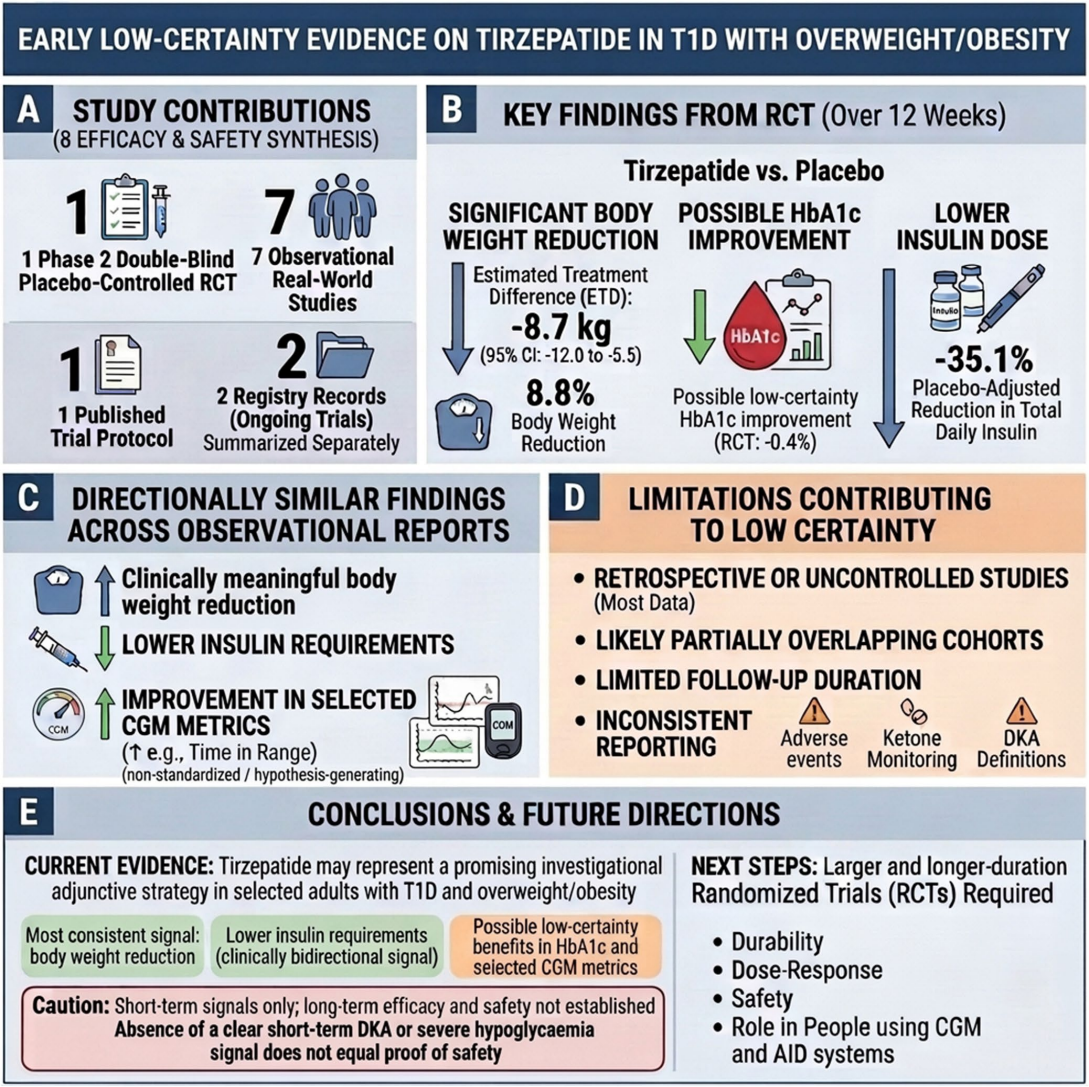
Visual synthesis of early low‐certainty evidence on tirzepatide in adults with type 1 diabetes and overweight or obesity. Panel A summarizes study contributions to the evidence base, including one phase 2 randomized placebo‐controlled trial, seven observational real‐world studies, one published trial protocol, and two ongoing registry records summarized separately. Panel B summarizes key findings from the randomized trial over 12 weeks, including significant body weight reduction, a possible low‐certainty HbA1c improvement, and lower insulin dose. Panel C summarizes directionally similar findings across observational reports, including clinically meaningful body weight reduction, lower insulin requirements, and improvement in selected CGM metrics. Panel D highlights major limitations contributing to low certainty, including retrospective or uncontrolled designs, likely partially overlapping cohorts, limited follow‐up duration, and inconsistent reporting of adverse events, ketone monitoring, and DKA definitions. Panel E summarizes overall conclusions and future research priorities, emphasizing that tirzepatide remains an investigational adjunctive strategy in selected adults with T1D and overweight or obesity and that larger, longer‐duration randomized trials are required.

These limitations are not incidental; they define the current state of the field. For that reason, the present review should be viewed primarily as a critical synthesis of early evidence rather than a basis for definitive therapeutic recommendations. A visual synthesis of the main findings, limitations, and future directions is provided in Figure 2.

## 5 Future Research Priorities

The available literature supports several priorities for future investigation. Larger and longer‐duration randomized trials are needed to determine the durability of effects on body weight, HbA1c, CGM outcomes, and insulin dose. Dose‐ranging evidence is required because the currently available randomized trial used only 2.5–5 mg weekly and does not establish outcomes at higher tolerated doses. Standardized safety monitoring, especially for ketones, DKA, severe hypoglycaemia, gastrointestinal intolerance, and discontinuation, is essential. Studies in AID users should evaluate how tirzepatide interacts with closed‐loop algorithms and define best practices for basal and bolus insulin adjustment. Patient‐selection studies should identify phenotypes most likely to benefit, including those with obesity, marked insulin resistance, high insulin requirements, or evidence of preserved endogenous insulin reserve. Comparative adjunctive studies should clarify how tirzepatide performs relative to or alongside other incretin‐based strategies already studied in T1D. Ongoing randomized studies, including TZP‐T1D and sponsor‐led trials, should be prioritized because they are likely to define the next phase of evidence generation.

## 6 Conclusions

The currently available evidence suggests that tirzepatide may represent a promising investigational adjunctive strategy for selected adults with T1D and overweight or obesity, particularly by promoting substantial body‐weight reduction. Early data also suggest possible benefit in HbA1c and selected CGM‐derived glucose metrics, and a reduction in insulin requirements is commonly observed. However, the evidence base remains preliminary and low‐certainty overall, with only one short‐term randomized trial and a predominance of non‐randomized studies at serious risk of bias.

At present, tirzepatide in T1D should be regarded as an emerging investigational strategy rather than an established therapy. Importantly, the absence of a consistent reported short‐term signal for severe hypoglycaemia or DKA should not be interpreted as proof of safety because ketone surveillance, event ascertainment, and DKA adjudication were not standardized across the available literature. The ultimate role of tirzepatide in T1D will depend on larger and longer‐duration randomized trials capable of defining long‐term efficacy, dose–response, safety, interactions with CGM and AID systems, and the patient subgroups most likely to benefit.

## Author Contributions

**Danilo Caponio:** conceptualization, investigation, funding acquisition, writing – original draft, writing – review and editing, visualization, validation, methodology, software, formal analysis, project administration, resources, supervision, data curation. **Giuseppina Alessia Acucella:** conceptualization, investigation, writing – original draft, funding acquisition, visualization, validation, methodology, formal analysis, project administration, resources, supervision, data curation.

## Funding

The authors have nothing to report.

## Conflicts of Interest

The authors declare no conflicts of interest.

## Supporting information


**Appendix S1:** Search strategy.

**Appendix S2:** Assessment of potential cohort overlap and implications for interpretation.

**Appendix S3:** Full‐text reports excluded (*n* = 14), with protocol‐defined reasons.

**Appendix S4:** GRADE Summary of Findings for tirzepatide as adjunct to insulin in adults with type 1 diabetes and overweight or obesity.

## Data Availability

The data that supports the findings of this study are available in the [Link], [Link], [Link], [Link] of this article.
