# Supplementary material for: Tirzepatide as Adjunct to Insulin in Adults With Type 1 Diabetes and Overweight or Obesity: A Systematic Review of Randomized and Real‐World Evidence
Source: Endocrinol Diabetes Metab. 2026 Apr 20;9(3):e70225. doi: 10.1002/edm2.70225 (PMC13093900; doi:10.1002/edm2.70225)
Supplement: Supplementary file 1 — Appendix S1: Search strategy. [file EDM2-9-e70225-s003.docx]

**Supplementary Appendix 1. Search strategy**

| **Database** | **Search string** | **Date of last search** |
| --- | --- | --- |
| **PubMed/MEDLINE** | (("Diabetes Mellitus, Type 1"[Mesh] OR "type 1 diabetes"[tiab] OR "type 1 diabetes mellitus"[tiab] OR T1D[tiab]) AND (tirzepatide[tiab] OR "Tirzepatide"[Mesh] OR LY3298176[tiab] OR Mounjaro[tiab] OR Zepbound[tiab] OR "dual GIP/GLP-1 receptor agonist"[tiab] OR "dual incretin agonist"[tiab] OR "GIP/GLP-1"[tiab] OR "glucose-dependent insulinotropic polypeptide receptor and glucagon-like peptide-1 receptor agonist"[tiab])) | 1 March 2026 |
| **Scopus** | TITLE-ABS-KEY("type 1 diabetes" OR "type 1 diabetes mellitus" OR T1D) AND (tirzepatide OR LY3298176 OR Mounjaro OR Zepbound OR “dual GIP/GLP-1 receptor agonist" OR "dual incretin agonist" OR "GIP/GLP-1" OR "glucose-dependent insulinotropic polypeptide receptor and glucagon-like peptide-1 receptor agonist")) | 1 March 2026 |
| **ClinicalTrials.gov** | Condition/Disease: Type 1 Diabetes OR Type 1 Diabetes Mellitus; Other terms: tirzepatide OR LY3298176 OR Mounjaro OR Zepbound | 1 March 2026 |
| **Embase (Ovid)** | ('type 1 diabetes mellitus'/exp OR 'type 1 diabetes':ti,ab OR 'type 1 diabetes mellitus':ti,ab OR t1d:ti,ab) AND (tirzepatide:ti,ab OR ly3298176:ti,ab OR mounjaro:ti,ab OR zepbound:ti,ab OR 'dual gip/glp-1 receptor agonist':ti,ab OR 'dual incretin agonist':ti,ab OR 'gip/glp-1':ti,ab OR 'glucose-dependent insulinotropic polypeptide receptor and glucagon-like peptide-1 receptor agonist':ti,ab) | 1 March 2026 |
| **Web of Science** | TS=(("type 1 diabetes" OR "type 1 diabetes mellitus" OR T1D) AND (tirzepatide OR LY3298176 OR Mounjaro OR Zepbound OR "dual GIP/GLP-1 receptor agonist" OR "dual incretin agonist" OR "GIP/GLP-1" OR "glucose-dependent insulinotropic polypeptide receptor and glucagon-like peptide-1 receptor agonist")) | 1 March 2026 |

**Supplementary Appendix 1. Search strategy.**

Full database search strategies used for PubMed/MEDLINE, Scopus, ClinicalTrials.gov, Embase (Ovid), and Web of Science. Searches were designed to maximize sensitivity for reports evaluating tirzepatide in adults with type 1 diabetes by combining controlled vocabulary and free-text terms for the population and intervention only. Developmental, mechanistic, and product-name variants for tirzepatide were included where applicable. Search syntax was adapted to each database platform. To reduce the risk of missing early-phase or variably indexed reports, the core strategy did not require obesity-, glycaemia-, insulin-, or safety-specific terms. The date of the last search for all sources was 1 March 2026.
