# Supplementary material for: Tirzepatide as Adjunct to Insulin in Adults With Type 1 Diabetes and Overweight or Obesity: A Systematic Review of Randomized and Real‐World Evidence
Source: Endocrinol Diabetes Metab. 2026 Apr 20;9(3):e70225. doi: 10.1002/edm2.70225 (PMC13093900; doi:10.1002/edm2.70225)
Supplement: Supplementary file 2 — Appendix S2: Assessment of potential cohort overlap and implications for interpretation. [file EDM2-9-e70225-s001.docx]

**Supplementary Appendix 2. Assessment of potential cohort overlap and implications for interpretation**

| **Study** | **Center / network** | **Publication or analytic context** | **Potential overlap with** | **Reason for concern** | **Interpretive implication** |
| --- | --- | --- | --- | --- | --- |
| Akturk et al. [10] | University of Colorado / related investigative network | Early proof-of-concept observational cohort | Garg et al. [11], Karakus et al. [12], Snell-Bergeon et al. [14], Garg et al. [15] | Same broad investigative environment; early clinical experience may have contributed to later Colorado-associated analyses | Should be interpreted as an early signal-generating cohort, not as fully independent confirmation relative to later Colorado-network reports |
| Garg et al. [11] | Colorado-associated network | 12-month matched observational comparative study | Akturk et al. [10], Karakus et al. [12], Snell-Bergeon et al. [14], Garg et al. [15] | Same regional/investigative network; treated cohort derived from a broader prescribed population; shared clinical source population cannot be excluded | Apparent consistency with related reports should not be interpreted as external replication |
| Karakus et al. [12] | Same investigative environment | AID-focused insulin-adjustment analysis | Akturk et al. [10], Garg et al. [11] | Authors state the analysis used data from a previously published tirzepatide study; likely subgroup or re-analysis of related source cohort | Best interpreted as a complementary AID-specific analysis rather than an independent cohort |
| Snell-Bergeon et al. [14] | Colorado-associated network | Comparative effectiveness analysis of semaglutide and tirzepatide | Akturk et al. [10], Garg et al. [11], Garg et al. [15] | Same clinical/research network; overlap in treated source population cannot be excluded | Comparative findings are informative, but not clearly independent of prior Colorado-network reports |
| Garg et al. [15] | Colorado-associated network | Long-term 21-month biomarker-focused matched analysis | Akturk et al. [10], Garg et al. [11], Snell-Bergeon et al. [14] | Same broad network; later analytic follow-up with different outcome emphasis may represent reconfiguration of a related treated cohort | Long-term results should be interpreted as an extension of a related observational experience, not as distinct external validation |
| Rivera Gutierrez et al. [13] | Mayo Clinic | Independent retrospective clinical cohort | None clearly identified | Different center and reporting structure | Overlap with Colorado-network reports appears unlikely |
| Al Ozairi et al. [16] | Separate real-world comparative cohort | 12-month comparative study of tirzepatide, semaglutide, and liraglutide | None clearly identified | Distinct geographic and clinical setting | Overlap with US center-based cohorts appears unlikely |

**Supplementary Appendix 2. Assessment of potential cohort overlap and implications for interpretation.**

Structured assessment of potential overlap among observational studies, including center/network, analytic context, potentially overlapping reports, reasons for concern, and interpretive implications. Several observational reports appear to arise from the same or related Colorado-associated clinical and investigative networks. Accordingly, the observational literature should be interpreted as showing directionally similar findings across reports, but not necessarily independent replication across distinct settings. This reduces confidence in any inference based on apparent cross-study consistency alone.
