# Supplementary material for: Tirzepatide as Adjunct to Insulin in Adults With Type 1 Diabetes and Overweight or Obesity: A Systematic Review of Randomized and Real‐World Evidence
Source: Endocrinol Diabetes Metab. 2026 Apr 20;9(3):e70225. doi: 10.1002/edm2.70225 (PMC13093900; doi:10.1002/edm2.70225)
Supplement: Supplementary file 3 — Appendix S3: Full‐text reports excluded (n = 14), with protocol‐defined reasons. [file EDM2-9-e70225-s004.docx]

**Supplementary Appendix 3. Full-text reports excluded (n = 14), with protocol-defined reasons**

| **Full-text report** | **Reason for exclusion** |
| --- | --- |
| Roche E, Chapon J, Bogenmann L, Luyton C. *Acta Diabetologica*. 2025. | Single-patient case report; excluded from efficacy synthesis |
| Mendoza F, Parsiani R. *J Am Pharm Assoc (2003).* 2023;63(6):1821-1825. | Single-patient case report; excluded from efficacy synthesis |
| Ahmed K, Yaqoob N, Qaisar Z, et al. *Journal of Diabetology.* 2024;15(4):447-451. | Single-patient case report; excluded from efficacy synthesis |
| Baretić M. *Diabetes Technol Ther.* 2025;27(11):960-961. | Letter / single-patient experience; no extractable cohort outcomes |
| Patoulias D, Imprialos K, Doumas M. *Eur J Intern Med.* 2026;145:106624. | Editorial/commentary; no original outcome data |
| Infante M, Spagnoli A, Bielawski K, et al. *J Clin Med.* 2025;14(4):1303. | Narrative review; no original outcome data |
| Dutta D, Minocha R, Jha A. *Diabetes Technology and Obesity Medicine.* 2025;1(1):289-298. | Review article; no new primary outcome data |
| Malik M, Khan R, Fisher G, et al. *Clin Case Rep.* 2026;14:e71929. | Single-patient case report of euDKA in T1D with concomitant SGLT2 inhibitor; excluded from efficacy synthesis, considered contextual safety signal only |
| Louwagie EJ, Diego D, et al. *JCEM Case Rep.* 2025;3(3):luaf028. | Wrong population / T1D data not separable |
| Sultan W, Spencer J. *Cureus.* 2025;17(6):e87031. | Wrong population (insulin-dependent type 2 diabetes) |
| Singh R. *Cureus.* 2025;17(4):e83187. | Wrong population (non-diabetic) |
| Campana C, Patel M, Tran MD, et al. *J Emerg Med.* 2026;83:57-60. | Wrong population / T1D data not separable |
| Pond K, Rees K, Nicholson I, et al. *JCEM Case Rep.* 2026;4(2):luaf324. | Wrong population / not T1D |
| Unpublished/grey literature record identified during full-text screening: “Cardiorenal benefit of tirzepatide in type 1 diabetes” | Insufficient bibliographic information / full text not retrievable; data extraction and risk-of-bias assessment not possible |

**Supplementary Appendix 3. Full-text reports excluded (n = 14), with protocol-defined reasons.**
List of full-text reports excluded after eligibility assessment, together with prespecified reasons for exclusion. Exclusion categories included single-patient case reports or case-based publications, review/editorial/commentary/letter articles without original cohort outcome data, wrong population or T1D data not separable, and insufficient bibliographic information or non-retrievable full text. One published trial protocol was retained separately to describe ongoing evidence generation and was not included in the efficacy and safety synthesis.
