# Supplementary material for: Tirzepatide as Adjunct to Insulin in Adults With Type 1 Diabetes and Overweight or Obesity: A Systematic Review of Randomized and Real‐World Evidence
Source: Endocrinol Diabetes Metab. 2026 Apr 20;9(3):e70225. doi: 10.1002/edm2.70225 (PMC13093900; doi:10.1002/edm2.70225)
Supplement: Supplementary file 4 — Appendix S4: GRADE Summary of Findings for tirzepatide as adjunct to insulin in adults with type 1 diabetes and overweight or obesity. [file EDM2-9-e70225-s002.docx]

**Supplementary Appendix 4. GRADE Summary of Findings for tirzepatide as adjunct to insulin in adults with type 1 diabetes and overweight or obesity**

| **Outcome** | **Studies contributing** | **Direction of observed effect** | **Certainty of evidence (GRADE)** | **Reasons for downgrading** |
| --- | --- | --- | --- | --- |
| **Body weight reduction** | 1 randomized trial + multiple observational studies | Consistent signal toward body weight reduction; strongest and most reproducible observed outcome | **Low** | Downgraded for **risk of bias** (single small RCT with some concerns; observational studies at serious risk of bias), **indirectness** (heterogeneous populations, dose escalation, follow-up), and **imprecision** (single small RCT; observational estimates not pooled) |
| **HbA1c** | 1 randomized trial + multiple observational studies | Possible improvement in HbA1c; magnitude potentially clinically meaningful, but short-term and not clearly independent of confounding/mediation | **Very low** | Downgraded for **risk of bias**, **imprecision**, **indirectness**, and concern that observed effects may reflect **weight loss, insulin retitration, technology use, or clinical attention** rather than a clearly independent drug effect |
| **Total daily insulin dose** | 1 randomized trial + multiple observational studies | Frequent reduction in insulin requirements across studies | **Very low** | Downgraded for **risk of bias**, **indirectness**, and **serious concerns about interpretability**, because insulin reduction was not protocolized, may be clinician-driven, and is a clinically bidirectional rather than uniformly beneficial endpoint |
| **CGM-derived metrics** (e.g., TIR, TAR, mean glucose) | Mainly observational studies; limited randomized evidence | Possible improvement in selected CGM metrics | **Very low** | Downgraded for **risk of bias**, **inconsistency in definitions/reporting**, **indirectness**, and **imprecision**; outcomes were non-standardized and should be considered hypothesis-generating |
| **Severe hypoglycaemia** | Randomized and observational reports | No clear increase reported, but evidence insufficient for reassurance | **Very low** | Downgraded for **very serious imprecision** (rare event, sparse data), **risk of bias**, and **inadequate event ascertainment/reporting** |
| **DKA / ketosis** | Randomized and observational reports; contextual case reports excluded from synthesis | No clear short-term signal in included studies, but clinically important risk cannot be excluded | **Very low** | Downgraded for **very serious imprecision**, **risk of bias**, **indirectness**, and **insufficient ketone surveillance / non-standardized DKA definitions**; absence of reported events does not establish safety |
| **Gastrointestinal adverse events** | Randomized and observational studies | Gastrointestinal adverse events were the most commonly reported adverse events | **Low** | Downgraded for **risk of bias** and **indirectness**; event ascertainment and severity grading were inconsistently reported, but a class-consistent signal was present across studies |
| **Treatment discontinuation** | Primarily observational studies; limited randomized reporting | Discontinuation occurred in some cohorts, but rates and reasons were inconsistently captured | **Very low** | Downgraded for **risk of bias**, **imprecision**, and **inconsistent reporting**; discontinuation was not systematically or uniformly described across studies |

**Supplementary Appendix 4. GRADE Summary of Findings for tirzepatide as adjunct to insulin in adults with type 1 diabetes and overweight or obesity.**

Structured certainty-of-evidence summary for the main efficacy and safety outcomes, including body weight reduction, HbA1c, total daily insulin dose, CGM-derived metrics, severe hypoglycaemia, DKA/ketosis, gastrointestinal adverse events, and treatment discontinuation. Certainty ratings were based on study design, within-study risk of bias, inconsistency, indirectness, imprecision, and publication/reporting limitations. Because no meta-analysis was performed, certainty judgments were based on structured qualitative synthesis of direction, coherence, and limitations of the evidence rather than pooled effect estimates. Several observational reports may represent partially overlapping cohorts from the same or related investigative networks; therefore, apparent consistency across observational studies should not be interpreted as equivalent to independent replication. For safety outcomes, especially DKA/ketosis and severe hypoglycaemia, absence of reported events in small or retrospectively observed cohorts was not considered evidence of safety. Insulin dose reduction was treated as a clinically ambivalent outcome in T1D because it may reflect improved metabolic efficiency but may also increase vulnerability to underinsulinization and ketosis if not carefully managed.
